# Supplementary material for: Carbon Quantum Dots Based Chemosensor Array for Monitoring Multiple Metal Ions
Source: Molecules. 2022 Jun 15;27(12):3843. doi: 10.3390/molecules27123843 (PMC9227453; doi:10.3390/molecules27123843)
Supplement: Supplementary file 1 [file molecules-27-03843-s001.zip › molecules-1742915-supplementary.pdf]

Supplementary Information

Carbon Quantum Dots based Chemosensor Array for Monitoring Multiple Metal Ions

Tianlei Qin [1], Jiayi Wang [1], Song Guo[1*] and Yuanli Liu[1*]

[1]Key Laboratory of New Processing Technology for Nonferrous Metal & Materials, Guilin University of Technology, Ministry of Education, Guilin 541004, China

*** Correspondence:**
Yuanli Liu: lyuanli@glut.edu.cn

Song Guo: bobingjin@glut.edu.cn

Supplementary Information Contents:

1 Supplemental experimental section

2 Optimization of detection conditions

3 Fluorescence titration of metal ions

4 Metal ion induced sensing element aggregation

5 Linear Discriminant Analysis (LDA)

6 Results of the Quantitative Assay

7 Blind Assay

1. Supplemental experimental section

Characterization of AC-CQDs

The activated carbon is treated by chemical oxidation method to obtain fluorescent AC-CQDs with good performance, and the morphology, structure and optical properties of the prepared AC-CQDs are characterized. The morphology of AC-CQDs is characterized by transmission electron microscopy. As shown in **Figure S1a**, AC-CQDs exhibit uniform shape and ultra-small size (average size around 3 nm), and the high-resolution TEM image in **Figure S1b** can observe a fine crystal structure consistent with the (020) lattice stripe of graphitic carbon [1], with a lattice stripe spacing of 0.23 nm. The XRD patterns of the AC-CQDs are also measured, as shown in **Figure S2a**, with a broad diffraction peak distributed around 25°, which is considered to be the (002) plane of graphitic carbon [2,3]. The surface groups of AC-CQDs are determined by Fourier Transform spectroscopy (FTIR) and X-ray photoelectron spectroscopy (XPS). As shown in the FTIR spectrum of **Figure S2b**, a distinct peak at 3403 cm^{-1} is attributed to the asymmetric vibration of the carboxyl group, the peaks at 2898 and 2958 cm^{-1} are related to the stretching vibration of C-H, and the four peaks at 1066, 1265, 1608 and 1712 cm^{-1} correspond to C-O, C-N, C=C and C=O, respectively. The XPS spectrum of AC-CQDs shows a strong C1s peak centered at 284.08 eV (**Figure S2c**), where the content of C is 80.29%. The high-resolution spectrum of C1s is shown in **Figure S2d** with four main peaks at 284.38 eV (C-C/C=C), 285.01 eV (C-N), 285.78 eV (C-O) and 288.38 eV (C-OOR), and the results are similar to FTIR. Among them, the binding energy peak at 284.38 eV corresponds to the graphite structure of carbon quantum dots (sp^2 C-C). These rich functional groups can undergo complex reactions with metal ions through hydrogen bonding and electrostatic interaction, which is the key to metal ion array detection. Meanwhile, As shown in **Figure S3**, the changes in the aqueous solutions of activated carbon (left) and AC-CQDs (right) at the time points of 1min, 10min, 1h, and 10h can prove that AC-CQDs have excellent dispersion and water solubility compared with activated carbon.

We dissolve the obtained dried solid of AC-CQDs in ultrapure water, and the fluorescence intensity gradually increases with increasing concentration of AC-CQDs until the concentration reached 0.15 mg/mL and then gradually weakened (**Figure S4**). The fluorescence quenching at high concentrations is attributed to aggregation-induced quenching, and AC-CQDs solutions are all performed at concentration of 0.15 mg/mL in the future. AC-CQDs show a similar excitation wavelength dependence to other reported carbon quantum dots [4-6], with the corresponding emission wavelength of AC-CQDs gradually shifting from 536 nm to 574 nm as the excitation wavelength increases from 300 nm to 500 nm (**Figure S5a**). This shift in emission wavelength is attributed to the presence of different functional groups and size effects on the surface of AC-CQDs, which lead to changes in fluorescence emission. As can be easily observed from the thermogram in **Figure S5b**, the optimal excitation wavelength occurs at 450 nm, corresponding to the maximum emission peak centered at 524 nm. AC-CQDs show a strong absorption peak near 285 nm, which is associated with the π-π^* transition of C=C or C=N. Due to the narrow size distribution of AC-CQDs, the solution exhibits a symmetrical emission spectrum and strong yellow-green fluorescence under ultraviolet light at 365 nm (**Figure S6a**). In practical applications, the complex environmental conditions have an important influence on the fluorescence stability of AC-CQDs. Therefore, the pH and salt concentration of the solution are necessary to investigate the fluorescence performance of AC-CQDs. As the pH of the AC-CQDs solution changes from 4 to 11, it exhibits relatively stable fluorescence emission intensity (**Figure S6b**). The effect of salt concentration on the fluorescence intensity of AC-CQDs is also investigated. As shown in **Figure S6c**, the fluorescence

intensity of AC-CQDs does not diminish and even slightly enhance when the concentration of sodium chloride reached 1 M. We also investigate the storage stability of AC-CQDs, it could observe that the fluorescence intensity of AC-CQDs slightly decrease in the first 1 h and remain essentially unchanged for the next 10 h (**Figure S6d**). Thus, the stable fluorescence properties of AC-CQDs provide a good platform for sensing and imaging applications. To assess whether AC-CQDs would have a toxic effect on humans, we adopt the methylthiazole diphenyl tetrazolium bromide (MTT) assay to detect its toxicity in Hela cells. As shown in **Figure S7**, the cell survival rate of AC-CQDs is close to 100% at concentrations below 50 μg/mL, and the cell survival rate is 60.5% even when the concentration of AC-CQDs reach 300 μg/mL, demonstrating the great potential of AC-CQDs for biological applications. We further investigate the ability of AC-CQDs for bioimaging. Confocal microscopy (CLSM) observe that cells incubated with 150 μg/mL AC-CQDs for 3 h maintain normal morphology (bright field), demonstrating the low toxicity of AC-CQDs, and cytoplasmic locations show a bright fluorescent signals, indicating that AC-CQDs are successfully accumulated by cellular internalization. The merger of fluorescence images and bright-field images suggests that the effective cell staining properties of AC-CQDs imply potential applications in bioimaging.

Calculation of fluorescence quantum yield (QY)

The fluorescence quantum yields (QY) of AC-CQDs and other sensing elements are calculated in aqueous solutions by a comparative method [7,8]. We chose quinine sulfate (0.1 M H2SO4 as solvent, QY=54%) as the reference standard to determine the fluorescence quantum yields of the sensing elements. A certain concentration of quinine sulfate solution is prepared in 0.1 M H2SO4, the absorbance value at the excitation wavelength of the maximum fluorescence emission of quinine sulfate is recorded (0.02 ~ 0.05), and measure the fluorescence of quinine sulfate and the sensor element at this excitation wavelength. QY is calculated according to the following equation,

$$\Phi_{f_x} = \frac{{n^2}_x}{{n^2}_{std}} \cdot \frac{A_{std} \cdot F_x}{A_x \cdot F_{std}} \cdot \Phi_{f_{std}}$$

where $\Phi_{f_{std}}$ is the fluorescence quantum yield (54%) of quinine sulfate (0.1 M H2SO4), A_{std} and A_x are the absorbance values of quinine sulfate and the sensing element at the excitation wavelength, respectively, F_{std} and F_x are the integrated areas of the fluorescence spectra of quinine sulfate and the sensing element at this excitation wavelength, respectively, and n is the refractive index of the solution.

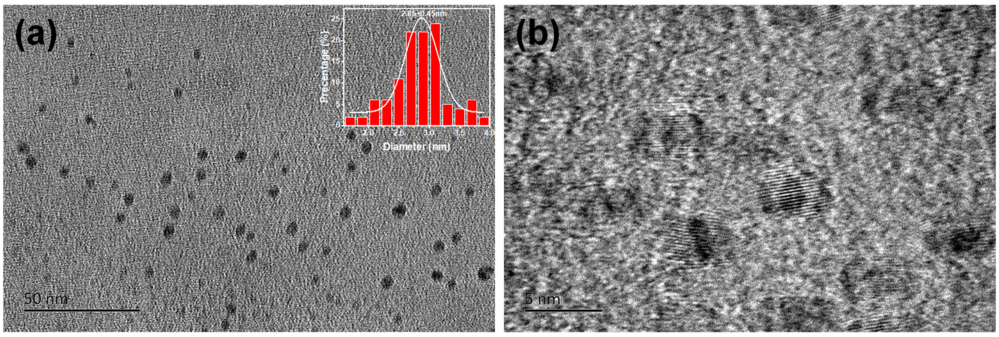

Figure S1. (a) The TEM image of AC-CQDs and size distribution of the CQDs (inset). (b) HR-TEM image of AC-CQDs.

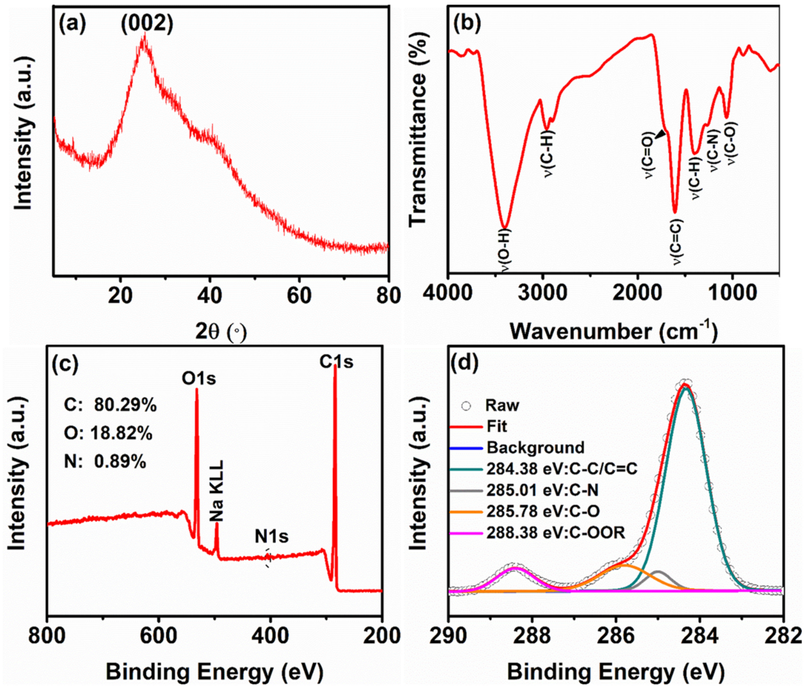

Figure S2. Characterization of AC-CQDs including: (a) XRD patterns of N-CQDs, (b) FTIR spectra, (c) XPS spectra, (d) High-resolution XPS C1s spectra.

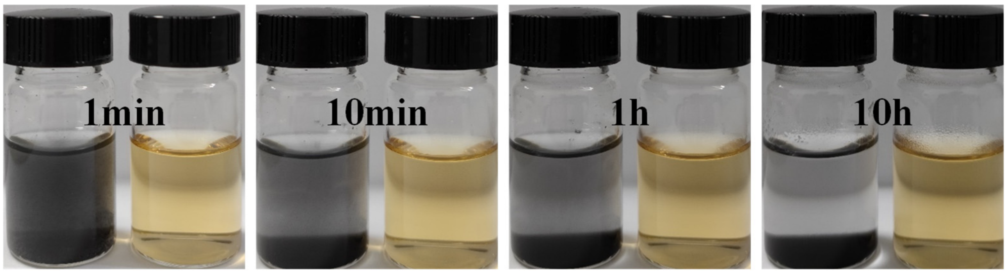

Figure S3. Photos of activated carbon solution (left) and synthesized AC-CQDs solution (right) under different time conditions.

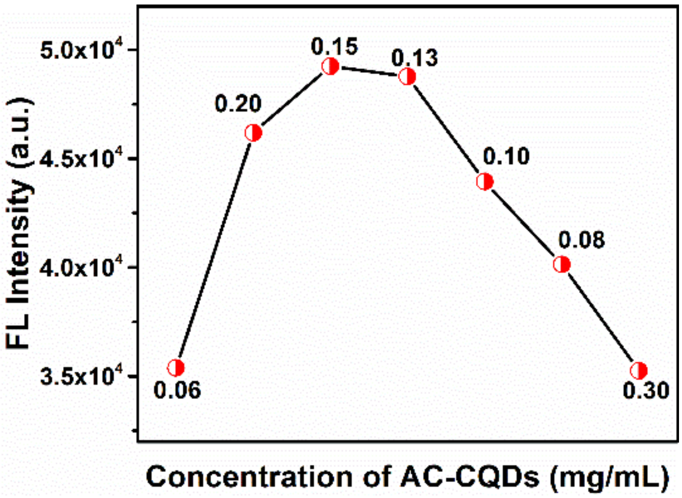

Figure S4. Effect of AC-CQDs concentration on fluorescence emission intensity.

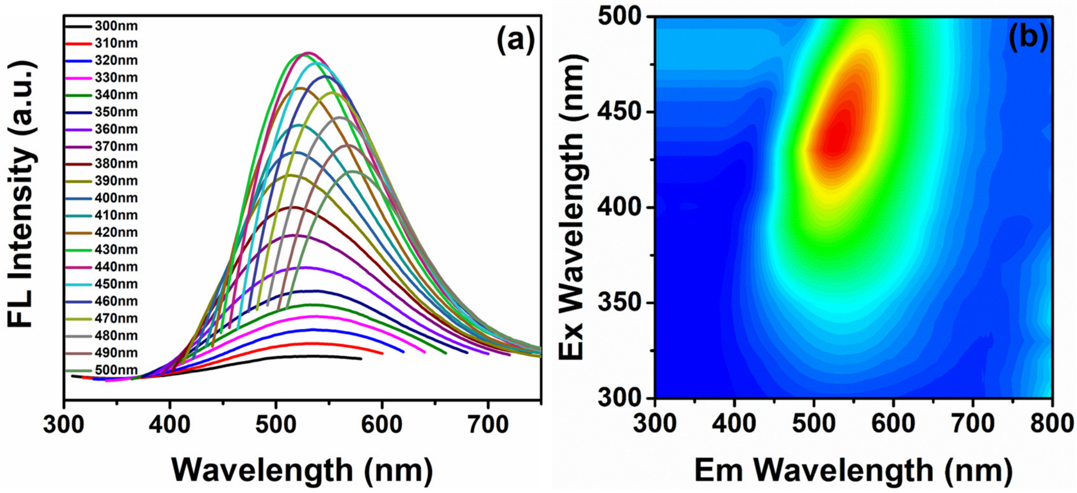

Figure S5. (a) Emission spectra of AC-CQDs solutions with increasing excitation wavelength from 300 nm to 500 nm. (b) Thermogram of the corresponding emission wavelengths at different excitation wavelengths.

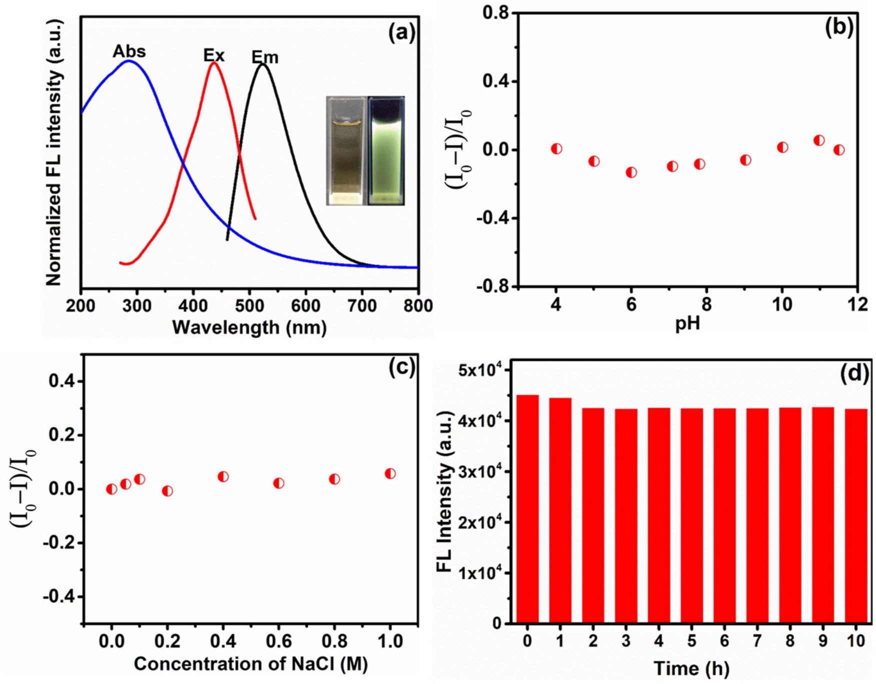

Figure S6. (a) UV-Vis absorption (Abs), fluorescence excitation (λ_{Ex} = 450 nm) and emission (λ_{Em} = 524 nm) spectra. The inset shows the photographs of AC-CQDs solutions under daylight and 365 nm UV light. (b) Effect of pH on the fluorescence intensity of AC-CQDs solution. (c) Effect of NaCl concentration on the fluorescence intensity of AC-CQDs solution. (d) Fluorescence stability of AC-CQDs within 10 h.

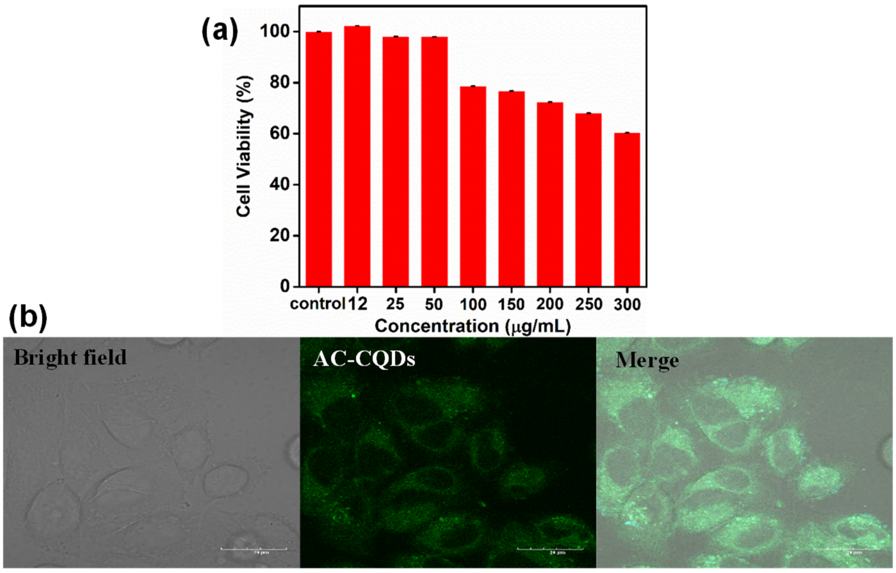

Figure S7. (a) Cell viability of Hela cells after 24 h incubation with different concentrations of AC-CQDs. CLSM bright field and fluorescence and merged images of Hela cells after 3 h incubation with AC-CQDs (150 μg/mL). Scale bar is 20 μm.

2. Optimization of detection conditions

Table S1. Quantum yields of different sensing elements.

QY (%)	CQDs	CQDs-Gln	CQDs-His	CQDs-Arg	CQDs-Lys	CQDs-Pro
	0.692	0.659	0.664	0.711	0.739	0.612

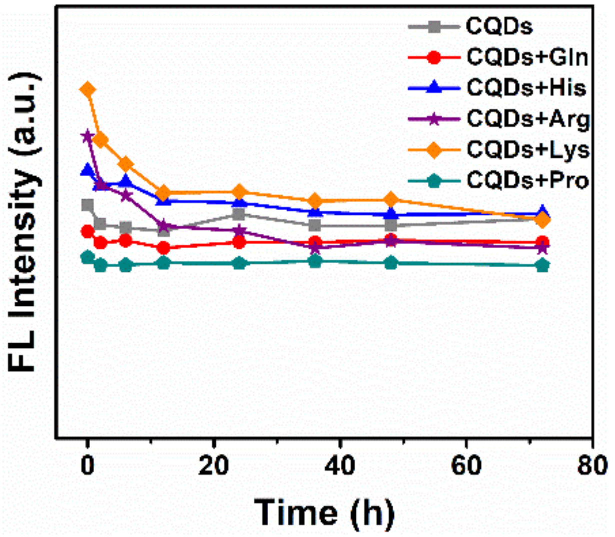

Figure S8. Fluorescence stability of different sensing elements within 72 h.

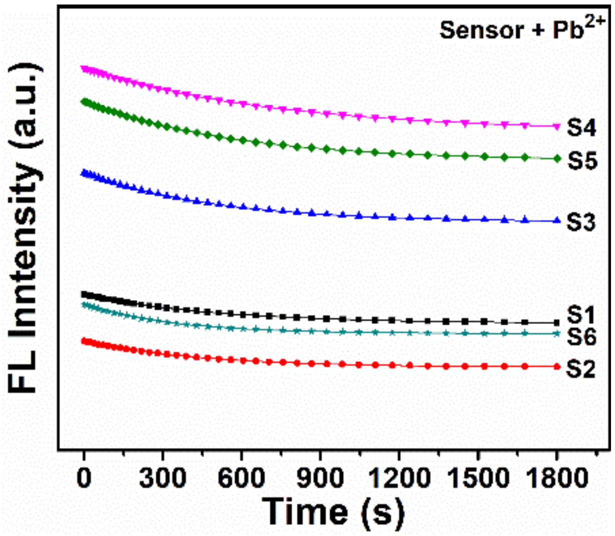

Figure S9. Effect of reaction time on the quenching efficiency of different sensing elements in a solution containing 100 μM Pb^{2+} at pH 5.0 (λ_{Ex}=450 nm, λ_{Em}=524 nm).

3. Fluorescence titration of metal ions

Table S2. Ksv calculation values of different sensing elements(mol/L).

	CQDs	CQDs-Gln	CQDs-His	CQDs-Arg	CQDs-Lys	CQDs-Pro
Mo^{5+}	2.38×10^3	2.71×10^3	2.14×10^3	2.21×10^3	3.22×10^3	3.04×10^3
Fe^{3+}	1.24×10^4	3.42×10^4	1.10×10^4	7.92×10^3	1.23×10^4	2.28×10^4
Cr^{3+}	8.69×10^3	1.64×10^4	1.70×10^4	1.94×10^4	2.02×10^4	1.32×10^4
Er^{3+}	1.09×10^4	3.06×10^4	3.47×10^4	3.54×10^4	3.68×10^4	3.23×10^4
Yb^{3+}	2.79×10^3	5.23×10^3	6.51×10^3	1.01×10^3	1.02×10^4	6.64×10^3
La^{3+}	3.83×10^3	7.14×10^3	9.60×10^3	8.24×10^3	1.08×10^4	8.65×10^3
Ni^{2+}	4.10×10^3	4.46×10^3	4.70×10^3	1.67×10^4	1.41×10^4	4.81×10^3
Cu^{2+}	7.43×10^3	1.35×10^4	1.10×10^4	3.56×10^4	3.21×10^4	1.47×10^4
Co^{2+}	4.46×10^3	6.30×10^3	8.70×10^3	1.81×10^4	1.94×10^4	6.28×10^3
Pb^{2+}	5.70×10^3	1.05×10^4	6.65×10^3	7.59×10^3	6.49×10^3	1.24×10^4
Mn^{2+}	1.71×10^3	2.92×10^3	7.09×10^3	1.08×10^4	1.01×10^4	3.05×10^3

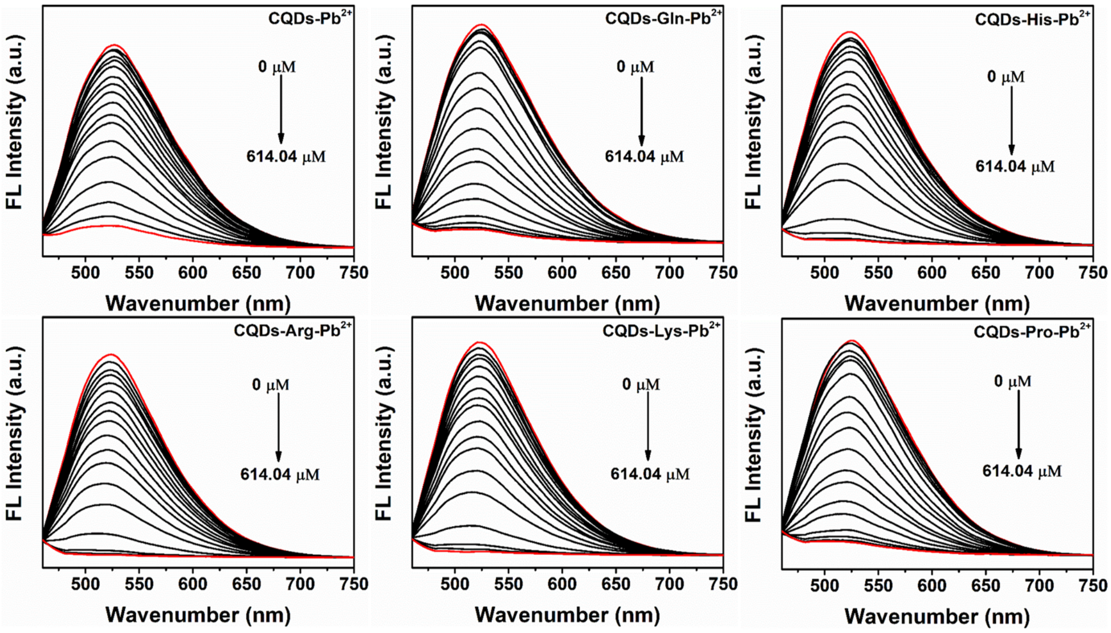

Figure S10. Photoluminescence spectra of six sensing elements at different Pb^{2+} concentrations.

4. Metal ion induced sensing element aggregation

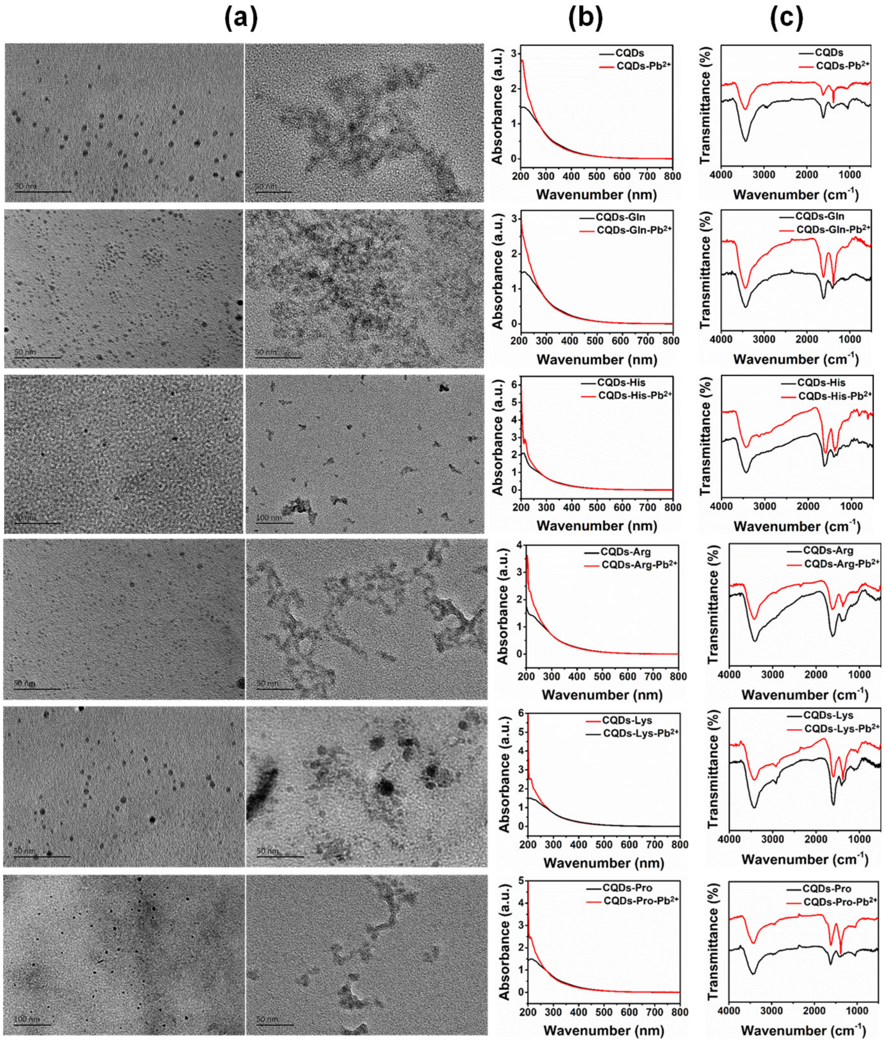

Figure S11. (a) TEM images of different sensing elements before and after the combination of Pb^{2+} (100 μM). (b) UV-Vis absorption spectra. (c) FTIR spectra.

Table S3. Fluorescence lifetime and Zeta potential values before and after the combination of different sensing elements with Pb^{2+} (100 μM).

	CQDs	CQDs-Pb^{2+}	CQDs-Gln	CQDs-Gln-Pb^{2+}	CQDs-His	CQDs-His-Pb^{2+}
Lifetime(ns)	2.12	2.24	2.40	2.45	2.10	2.24
Zeta(mV)	-35.5	-33.2	-33.1	-33.6	-37.8	-38.6
	CQDs-Arg	CQDs-Arg-Pb^{2+}	CQDs-Lys	CQDs-Lys-Pb^{2+}	CQDs-Pro	CQDs-Pro-Pb^{2+}
Lifetime(ns)	2.42	2.47	2.34	2.35	2.21	2.57
Zeta(mV)	-45.8	-42.7	-46.6	-42.8	-35.6	-34.5

5. Linear Discriminant Analysis (LDA)

Table S4. The jackknifed classification matrix of qualitative assay.

	Co^{2+}	Cr^{3+}	Cu^{2+}	Er^{3+}	Fe^{3+}	La^{3+}	Mn^{2+}	Mo^{5+}	Ni^{2+}	Pb^{2+}	Yb^{3+}	blank	%correct
Co^{2+}	20	0	0	0	0	0	0	0	0	0	0	0	100
Cr^{3+}	0	20	0	0	0	0	0	0	0	0	0	0	100
Cu^{2+}	0	0	20	0	0	0	0	0	0	0	0	0	100
Er^{3+}	0	0	0	20	0	0	0	0	0	0	0	0	100
Fe^{3+}	0	0	0	0	20	0	0	0	0	0	0	0	100
La^{3+}	0	0	0	0	0	20	0	0	0	0	0	0	100
Mn^{2+}	0	0	0	0	0	0	20	0	0	0	0	0	100
Mo^{5+}	0	0	0	0	0	0	0	20	0	0	0	0	100
Ni^{2+}	0	0	0	0	0	0	0	0	20	0	0	0	100
Pb^{2+}	0	0	0	0	0	0	0	0	0	20	0	0	100
Yb^{3+}	0	0	0	0	0	0	0	0	0	0	20	0	100
blank	0	0	0	0	0	0	0	0	0	0	0	20	100
Total	20	20	20	20	20	20	20	20	20	20	20	20	100

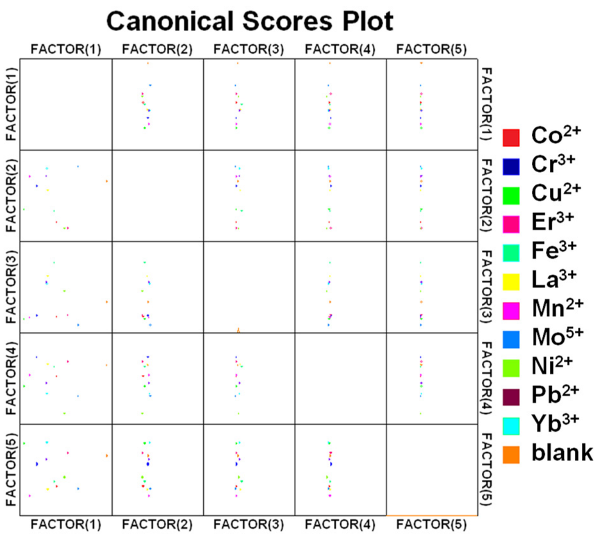

Figure S12. The canonical score plot of quantitative assay.

Table S5. The jackknifed classification matrix of semi-quantitative assay.

	Cr-40μM	Cr-60μM	Cr-80μM	Cr-100μM	Mn-5μM	Mn-10μM	Mn-20μM	Mn-40μM	Mn-60μM	Mn-80μM	Mn-100μM
Cr-40μM	20	0	0	0	0	0	0	0	0	0	0
Cr-60μM	0	20	0	0	0	0	0	0	0	0	0
Cr-80μM	0	0	20	0	0	0	0	0	0	0	0
Cr-100μM	0	0	0	20	0	0	0	0	0	0	0
Mn-5μM	0	0	0	0	20	0	0	0	0	0	0
Mn-10μM	0	0	0	0	0	20	0	0	0	0	0
Mn-20μM	0	0	0	0	0	0	20	0	0	0	0
Mn-40μM	0	0	0	0	0	0	0	20	0	0	0
Mn-60μM	0	0	0	0	0	0	0	0	20	0	0
Mn-80μM	0	0	0	0	0	0	0	0	0	20	0
Mn-100μM	0	0	0	0	0	0	0	0	0	0	20
Pb-5μM	0	0	0	0	0	0	0	0	0	0	0
Pb-10μM	0	0	0	0	0	0	0	0	0	0	0
Pb-20μM	0	0	0	0	0	0	0	0	0	0	0
Pb-40μM	0	0	0	0	0	0	0	0	0	0	0
Pb-60μM	0	0	0	0	0	0	0	0	0	0	0
Pb-80μM	0	0	0	0	0	0	0	0	0	0	0
Pb-100μM	0	0	0	0	0	0	0	0	0	0	0
blank	0	0	0	0	0	0	0	0	0	0	0
Total	20	20	20	20	20	20	20	20	20	20	20

Jackknifed Classification Matrix (contd...)

	Pb-5μM	Pb-10μM	Pb-20μM	Pb-40μM	Pb-60μM	Pb-80μM	Pb-100μM	blank	%correct
Cr-40μM	0	0	0	0	0	0	0	0	100
Cr-60μM	0	0	0	0	0	0	0	0	100
Cr-80μM	0	0	0	0	0	0	0	0	100
Cr-100μM	0	0	0	0	0	0	0	0	100

**Mn-5μM**	**0**	**0**	**0**	**0**	**0**	**0**	**0**	**0**	**100**
**Mn-10μM**	**0**	**0**	**0**	**0**	**0**	**0**	**0**	**0**	**100**
**Mn-20μM**	**0**	**0**	**0**	**0**	**0**	**0**	**0**	**0**	**100**
**Mn-40μM**	**0**	**0**	**0**	**0**	**0**	**0**	**0**	**0**	**100**
**Mn-60μM**	**0**	**0**	**0**	**0**	**0**	**0**	**0**	**0**	**100**
**Mn-80μM**	**0**	**0**	**0**	**0**	**0**	**0**	**0**	**0**	**100**
**Mn-100μM**	**0**	**0**	**0**	**0**	**0**	**0**	**0**	**0**	**100**
**Pb-5μM**	**20**	**0**	**0**	**0**	**0**	**0**	**0**	**0**	**100**
**Pb-10μM**	**0**	**20**	**0**	**0**	**0**	**0**	**0**	**0**	**100**
**Pb-20μM**	**0**	**0**	**20**	**0**	**0**	**0**	**0**	**0**	**100**
**Pb-40μM**	**0**	**0**	**0**	**20**	**0**	**0**	**0**	**0**	**100**
**Pb-60μM**	**0**	**0**	**0**	**0**	**20**	**0**	**0**	**0**	**100**
**Pb-80μM**	**0**	**0**	**0**	**0**	**0**	**20**	**0**	**0**	**100**
**Pb-100μM**	**0**	**0**	**0**	**0**	**0**	**0**	**20**	**0**	**100**
**blank**	**0**	**0**	**0**	**0**	**0**	**0**	**0**	**20**	**100**
**Total**	**20**	**20**	**20**	**20**	**20**	**20**	**20**	**20**	**100**

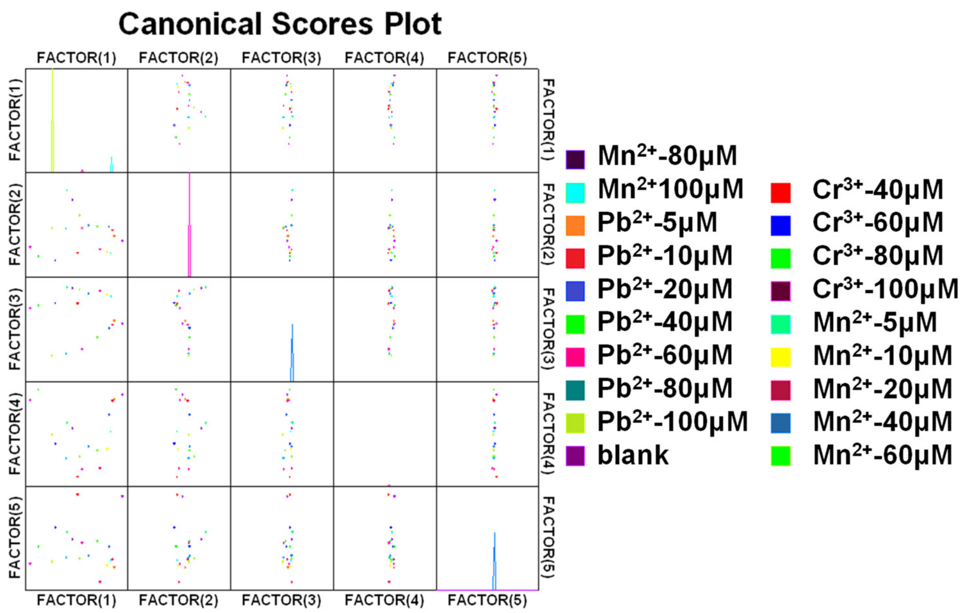

Figure S13. The canonical score plot of semi-quantitative assay.

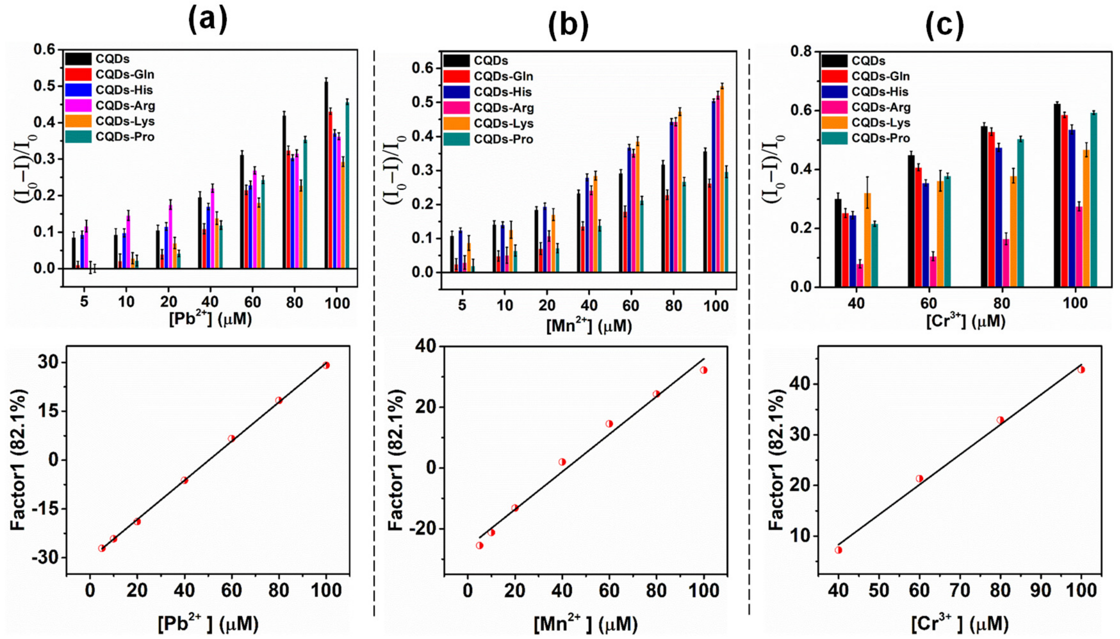

Figure S14. Different concentrations of metal ions (Pb^{2+}, Mn^{2+} and Cr^{3+}) are differentiated by sensor array system, corresponding to the fluorescence response pattern fingerprint and the linearity plot of the discriminant factor 1 against different metal ion concentrations ([Pb^{2+} and Mn^{2+}] = 5 ~ 100 μM, [Cr^{3+}] = 40 ~ 100 μM), respectively.

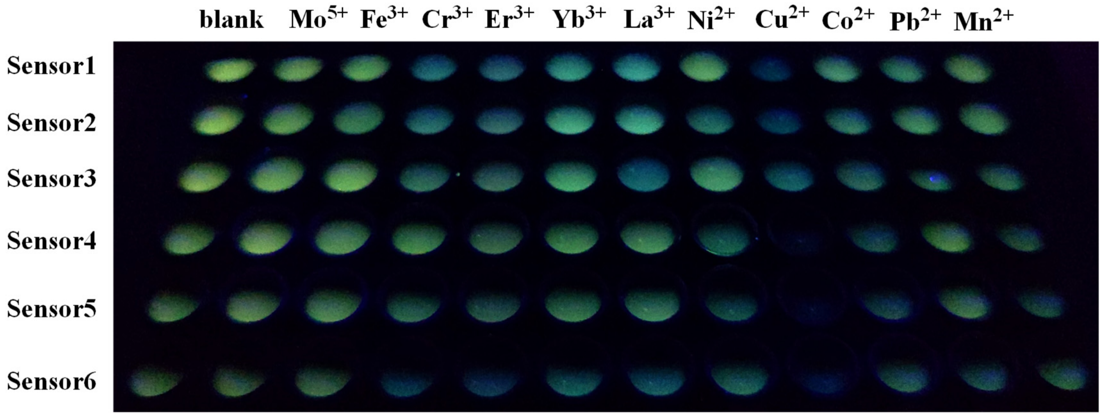

Figure S15. Photograph of self-assembled carbon quantum dot sensor array at room temperature, pH 5.0, and the presence or absence of metal ions. [AC-CQDs] = 0.15 mg/mL, [amino acid] = 500 μM, [metal ion] = 100 μM.

6. Results of the Quantitative Assay

Table S6. Mixed concentration conditions for metal ions in quantitative analysis at room temperature and pH 5.0. Red and gray correspond to the validation and calibration data sets, respectively.

Pb^{2+} (μM)	Mn^{2+} (μM)	Cr^{3+} (μM)
5	5	5
10	10	10
20	20	20
30	30	30
40	40	40
50	50	50
60	60	60
70	70	70
80	80	80
90	90	90
100	100	100

Table S7. Output calibration / Prediction data set of the quantitative assay for Pb^{2+} in the mixtures.

Pb^{2+}			
Calibration data set		Prediction data set	
Actual Pb^{2+} (μM)	Predicted Pb^{2+} (μM)	Actual Pb^{2+} (μM)	Predicted Pb^{2+} (μM)
5	5.12712	30	30.90331
5	5.090542	30	30.96836
5	5.009806	30	30.94247
5	5.30349	30	30.79704
5	5.301591	30	30.90259
5	4.999602	30	30.94494
5	5.085326	30	30.77135
5	5.082667	30	30.93535

5	5.024009	30	31.02684
5	5.043446	30	30.94988
5	5.001479	30	30.91974
5	5.109702	30	30.79047
5	4.98072	30	30.83056
5	5.007133	30	30.68756
5	5.059092	30	30.98858
5	4.975769	30	30.91918
5	5.213141	30	30.96982
5	5.041455	30	30.73758
5	4.928757	30	30.96266
5	5.261878	30	31.13309
10	10.14506	90	89.69612
10	9.98776	90	89.67758
10	9.954894	90	89.50975
10	9.683484	90	89.39742
10	9.740438	90	89.15843
10	9.781365	90	89.48437
10	9.856955	90	89.39418
10	9.958004	90	89.54327
10	10.3135	90	89.32882
10	9.819103	90	89.48324
10	9.880587	90	89.53272
10	9.849849	90	89.70277
10	10.11476	90	89.14252
10	9.992988	90	88.92868
10	9.851593	90	89.34201
10	10.04784	90	89.29751

10	9.923453	90	89.35968
10	9.837788	90	89.50292
10	9.854258	90	89.50069
10	10.01564	90	89.6081
20	19.91315		
20	19.90585		
20	19.77768		
20	19.93509		
20	20.14927		
20	20.13587		
20	20.31538		
20	19.94846		
20	20.10714		
20	20.00721		
20	19.72055		
20	19.86554		
20	19.94384		
20	20.13214		
20	20.06869		
20	19.97583		
20	19.86475		
20	19.92305		
20	19.9923		
20	19.95456		
40	40.02643		
40	39.96185		
40	40.31896		
40	39.76337		

**40**	**40.3205**
**40**	**39.88199**
**40**	**39.91392**
**40**	**40.31684**
**40**	**40.02216**
**40**	**39.87265**
**40**	**40.20856**
**40**	**40.02666**
**40**	**40.05849**
**40**	**39.96548**
**40**	**39.69483**
**40**	**40.12793**
**40**	**40.13616**
**40**	**39.82615**
**40**	**39.92684**
**40**	**39.86351**
**50**	**50.38068**
**50**	**49.99868**
**50**	**50.14272**
**50**	**50.26883**
**50**	**49.69316**
**50**	**49.89872**
**50**	**50.06416**
**50**	**50.06762**
**50**	**50.26763**
**50**	**49.79093**
**50**	**50.02329**
**50**	**50.14159**

50	50.10925
50	49.98892
50	49.96064
50	50.02484
50	50.3107
50	50.12001
50	50.14958
50	50.01702
60	59.75095
60	59.67285
60	59.83937
60	59.76374
60	59.77491
60	59.88908
60	59.76286
60	59.67439
60	60.05487
60	59.69206
60	59.78434
60	59.68003
60	59.99409
60	60.16393
60	59.89352
60	59.97063
60	60.01277
60	59.67693
60	59.82725
60	59.91974

70	70.13669
70	70.0546
70	70.1169
70	70.13602
70	70.00476
70	70.16435
70	70.09307
70	70.26722
70	70.24514
70	70.22509
70	70.31127
70	70.21702
70	70.42891
70	69.98546
70	70.17964
70	69.8119
70	70.02655
70	70.16583
70	70.10056
70	70.1094
80	80.23243
80	80.12265
80	80.19765
80	79.96399
80	79.9561
80	80.12547
80	79.91283
80	80.03742

80	80.0327
80	80.30344
80	80.24237
80	79.93841
80	79.73096
80	79.65089
80	80.16012
80	79.67813
80	79.92092
80	80.10036
80	79.92063
80	79.98823
100	99.97265
100	100.064
100	99.90611
100	100.0879
100	99.99156
100	99.69804
100	100.0863
100	99.78384
100	100.1287
100	99.97328
100	99.87555
100	100.1504
100	99.81603
100	100.2493
100	100.0216
100	99.92254

**100**	**99.7275**
**100**	**100.0171**
**100**	**100.3153**
**100**	**99.95927**

Table S8. Output calibration / Prediction data set of the quantitative assay for Mn^{2+} in the mixtures.

**Mn^{2+}**			
**Calibration data set**		**Prediction data set**	
**Actual Mn^{2+} (μM)**	**Predicted Mn^{2+} (μM)**	**Actual Mn^{2+} (μM)**	**Predicted Mn^{2+} (μM)**
**5**	**5.12712**	**30**	**30.90331**
**5**	**5.090542**	**30**	**30.96836**
**5**	**5.009806**	**30**	**30.94247**
**5**	**5.30349**	**30**	**30.79704**
**5**	**5.301591**	**30**	**30.90259**
**5**	**4.999602**	**30**	**30.94494**
**5**	**5.085326**	**30**	**30.77135**
**5**	**5.082667**	**30**	**30.93535**
**5**	**5.024009**	**30**	**31.02684**
**5**	**5.043446**	**30**	**30.94988**
**5**	**5.001479**	**30**	**30.91974**
**5**	**5.109702**	**30**	**30.79047**
**5**	**4.98072**	**30**	**30.83056**
**5**	**5.007133**	**30**	**30.68756**
**5**	**5.059092**	**30**	**30.98858**
**5**	**4.975769**	**30**	**30.91918**
**5**	**5.213141**	**30**	**30.96982**
**5**	**5.041455**	**30**	**30.73758**
**5**	**4.928757**	**30**	**30.96266**

5	5.261878	30	31.13309
10	10.14506	90	89.69612
10	9.98776	90	89.67758
10	9.954894	90	89.50975
10	9.683484	90	89.39742
10	9.740438	90	89.15843
10	9.781365	90	89.48437
10	9.856955	90	89.39418
10	9.958004	90	89.54327
10	10.3135	90	89.32882
10	9.819103	90	89.48324
10	9.880587	90	89.53272
10	9.849849	90	89.70277
10	10.11476	90	89.14252
10	9.992988	90	88.92868
10	9.851593	90	89.34201
10	10.04784	90	89.29751
10	9.923453	90	89.35968
10	9.837788	90	89.50292
10	9.854258	90	89.50069
10	10.01564	90	89.6081
20	19.91315		
20	19.90585		
20	19.77768		
20	19.93509		
20	20.14927		
20	20.13587		
20	20.31538		

20	19.94846
20	20.10714
20	20.00721
20	19.72055
20	19.86554
20	19.94384
20	20.13214
20	20.06869
20	19.97583
20	19.86475
20	19.92305
20	19.9923
20	19.95456
40	40.02643
40	39.96185
40	40.31896
40	39.76337
40	40.3205
40	39.88199
40	39.91392
40	40.31684
40	40.02216
40	39.87265
40	40.20856
40	40.02666
40	40.05849
40	39.96548
40	39.69483

40	40.12793
40	40.13616
40	39.82615
40	39.92684
40	39.86351
50	50.38068
50	49.99868
50	50.14272
50	50.26883
50	49.69316
50	49.89872
50	50.06416
50	50.06762
50	50.26763
50	49.79093
50	50.02329
50	50.14159
50	50.10925
50	49.98892
50	49.96064
50	50.02484
50	50.3107
50	50.12001
50	50.14958
50	50.01702
60	59.75095
60	59.67285
60	59.83937

60	59.76374
60	59.77491
60	59.88908
60	59.76286
60	59.67439
60	60.05487
60	59.69206
60	59.78434
60	59.68003
60	59.99409
60	60.16393
60	59.89352
60	59.97063
60	60.01277
60	59.67693
60	59.82725
60	59.91974
70	70.13669
70	70.0546
70	70.1169
70	70.13602
70	70.00476
70	70.16435
70	70.09307
70	70.26722
70	70.24514
70	70.22509
70	70.31127

70	70.21702
70	70.42891
70	69.98546
70	70.17964
70	69.8119
70	70.02655
70	70.16583
70	70.10056
70	70.1094
80	80.23243
80	80.12265
80	80.19765
80	79.96399
80	79.9561
80	80.12547
80	79.91283
80	80.03742
80	80.0327
80	80.30344
80	80.24237
80	79.93841
80	79.73096
80	79.65089
80	80.16012
80	79.67813
80	79.92092
80	80.10036
80	79.92063

80	79.98823
100	99.97265
100	100.064
100	99.90611
100	100.0879
100	99.99156
100	99.69804
100	100.0863
100	99.78384
100	100.1287
100	99.97328
100	99.87555
100	100.1504
100	99.81603
100	100.2493
100	100.0216
100	99.92254
100	99.7275
100	100.0171
100	100.3153
100	99.95927

Table S9 Output calibration / Prediction data set of the quantitative assay for Cr^{3+} in the mixtures.

Cr^{3+}			
Calibration data set		Prediction data set	
Actual Cr^{3+} (μM)	Predicted Cr^{3+} (μM)	Actual Cr^{3+} (μM)	Predicted Cr^{3+} (μM)
5	5.12712	30	30.90331
5	5.090542	30	30.96836

5	5.009806	30	30.94247
5	5.30349	30	30.79704
5	5.301591	30	30.90259
5	4.999602	30	30.94494
5	5.085326	30	30.77135
5	5.082667	30	30.93535
5	5.024009	30	31.02684
5	5.043446	30	30.94988
5	5.001479	30	30.91974
5	5.109702	30	30.79047
5	4.98072	30	30.83056
5	5.007133	30	30.68756
5	5.059092	30	30.98858
5	4.975769	30	30.91918
5	5.213141	30	30.96982
5	5.041455	30	30.73758
5	4.928757	30	30.96266
5	5.261878	30	31.13309
10	10.14506	90	89.69612
10	9.98776	90	89.67758
10	9.954894	90	89.50975
10	9.683484	90	89.39742
10	9.740438	90	89.15843
10	9.781365	90	89.48437
10	9.856955	90	89.39418
10	9.958004	90	89.54327
10	10.3135	90	89.32882
10	9.819103	90	89.48324

10	9.880587	90	89.53272
10	9.849849	90	89.70277
10	10.11476	90	89.14252
10	9.992988	90	88.92868
10	9.851593	90	89.34201
10	10.04784	90	89.29751
10	9.923453	90	89.35968
10	9.837788	90	89.50292
10	9.854258	90	89.50069
10	10.01564	90	89.6081
20	19.91315		
20	19.90585		
20	19.77768		
20	19.93509		
20	20.14927		
20	20.13587		
20	20.31538		
20	19.94846		
20	20.10714		
20	20.00721		
20	19.72055		
20	19.86554		
20	19.94384		
20	20.13214		
20	20.06869		
20	19.97583		
20	19.86475		
20	19.92305		

20	19.9923
20	19.95456
40	40.02643
40	39.96185
40	40.31896
40	39.76337
40	40.3205
40	39.88199
40	39.91392
40	40.31684
40	40.02216
40	39.87265
40	40.20856
40	40.02666
40	40.05849
40	39.96548
40	39.69483
40	40.12793
40	40.13616
40	39.82615
40	39.92684
40	39.86351
50	50.38068
50	49.99868
50	50.14272
50	50.26883
50	49.69316
50	49.89872

**50**	**50.06416**
**50**	**50.06762**
**50**	**50.26763**
**50**	**49.79093**
**50**	**50.02329**
**50**	**50.14159**
**50**	**50.10925**
**50**	**49.98892**
**50**	**49.96064**
**50**	**50.02484**
**50**	**50.3107**
**50**	**50.12001**
**50**	**50.14958**
**50**	**50.01702**
**60**	**59.75095**
**60**	**59.67285**
**60**	**59.83937**
**60**	**59.76374**
**60**	**59.77491**
**60**	**59.88908**
**60**	**59.76286**
**60**	**59.67439**
**60**	**60.05487**
**60**	**59.69206**
**60**	**59.78434**
**60**	**59.68003**
**60**	**59.99409**
**60**	**60.16393**

60	59.89352
60	59.97063
60	60.01277
60	59.67693
60	59.82725
60	59.91974
70	70.13669
70	70.0546
70	70.1169
70	70.13602
70	70.00476
70	70.16435
70	70.09307
70	70.26722
70	70.24514
70	70.22509
70	70.31127
70	70.21702
70	70.42891
70	69.98546
70	70.17964
70	69.8119
70	70.02655
70	70.16583
70	70.10056
70	70.1094
80	80.23243
80	80.12265

80	80.19765
80	79.96399
80	79.9561
80	80.12547
80	79.91283
80	80.03742
80	80.0327
80	80.30344
80	80.24237
80	79.93841
80	79.73096
80	79.65089
80	80.16012
80	79.67813
80	79.92092
80	80.10036
80	79.92063
80	79.98823
100	99.97265
100	100.064
100	99.90611
100	100.0879
100	99.99156
100	99.69804
100	100.0863
100	99.78384
100	100.1287
100	99.97328

**100**	**99.87555**
**100**	**100.1504**
**100**	**99.81603**
**100**	**100.2493**
**100**	**100.0216**
**100**	**99.92254**
**100**	**99.7275**
**100**	**100.0171**
**100**	**100.3153**
**100**	**99.95927**

7. Blind Assay

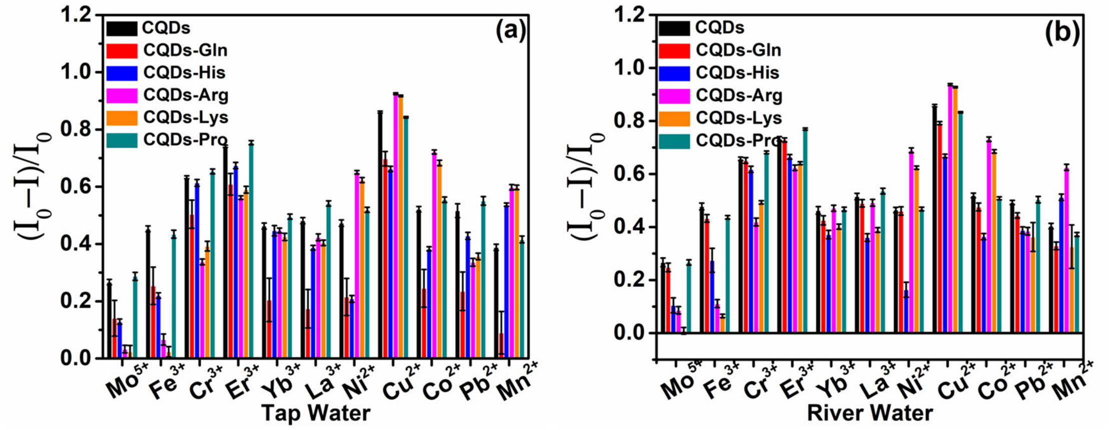

Figure S16. Fluorescence response pattern fingerprint of the sensor array in the presence of metal ions in different simulated environments: (a) tap water; (b) river water.

References

[1] Nie, H., Li, M., Li, Q., Liang, S., Tan, Y., Sheng, L., Shi, W., Zhang, S. (2014). Carbon dots with continuously tunable full-color emission and their application in ratiometric pH sensing. *Chem. Mat.* 26, 3104-3112. doi: 10.1021/cm5028157

[2] Liu, Y., Liu, Y., Park, S. J., Zhang, Y., Kim, T., Chae, S., Park, M., Kim, H. Y. (2015). One-step synthesis of robust nitrogen-doped carbon dots: acid-evoked fluorescence enhancement and their application in Fe^{3+} detection. *J. Mater. Chem.*

A. 3, 17747-17754. doi: 10.1039/c5ta05189d

[3] Lu, M., Duan, Y., Song, Y., Tan, J., Zhou, L. (2018). Green preparation of versatile nitrogen-doped carbon quantum dots from watermelon juice for cell imaging, detection of Fe^{3+} ions and cysteine, and optical thermometry. *J. Mol. Liq*. 269, 766-774. doi: 10.1016/j.molliq.2018.08.101

[4] Liao, J., Cheng, Z., Zhou, L. (2016). Nitrogen-doping enhanced fluorescent carbon dots: green synthesis and their applications for bioimaging and label-free detection of Au^{3+} ions. *ACS Sustain. Chem. Eng.* 4, 3053-3061. doi: 10.1021/acssuschemeng.6b00018

[5] Wang, R., Wang, X., Sun, Y. (2017). One-step synthesis of self-doped carbon dots with highly photoluminescence as multifunctional biosensors for detection of iron ions and pH. *Sens. Actuator B-Chem.* 241, 73-79. doi: 10.1016/j.snb.2016.10.043

[6] Yuan, Y., Liu, Z., Li, R., Zou, H., Lin, M., Liu, H., Huang, C. (2016). Synthesis of nitrogen-doping carbon dots with different photoluminescence properties by controlling the surface states. *Nanoscale.* 8, 6770-6776. doi: 10.1039/c6nr00402d

[7] Yang, G., Wan, X., Su, Y., Zeng, X., Tang, J. (2016). Acidophilic S-doped carbon quantum dots derived from cellulose fibers and their fluorescence sensing performance for metal ions in an extremely strong acid environment. *J. Mater. Chem. A*. 4, 12841-12849. doi: 10.1039/c6ta05943k

[8] Luo, T., Bu, L., Peng, S., Zhang, Y., Zhou, Z., Li, G., Huang, J. (2019). One-step microwave-assisted preparation of oxygen-rich multifunctional carbon quantum dots and their application for Cu^{2+}-curcumin detection. *Talanta.* 205, 120117. doi: 10.1016/j.talanta.2019.120117
